# The Utility of Predictive Modeling and a Systems Process Approach to Reduce Emergency Department Crowding: A Position Paper

**DOI:** 10.2196/42016

**Published:** 2023-07-10

**Authors:** Ann Corneille Monahan, Sue S Feldman

**Affiliations:** 1 University of Alabama at Birmingham Birmingham, AL United States; 2 Department of Health Services Administration University of Alabama at Birmingham Birmingham, AL United States

**Keywords:** emergency care, prehospital, information systems, crowding, healthcare service, healthcare system, emergency department, boarding, exit block, medical informatics, application, health services research, personalized medicine, predictive medicine, model, probabilistic, polynomial model, decision support technique, systems approach, predict, evidence based health care, hospital bed management, management information systems, position paper

## Abstract

Emergency department (ED) crowding and its main causes, exit block and boarding, continue to threaten the quality and safety of ED care. Most interventions to reduce crowding have not been comprehensive or system solutions, only focusing on part of the care procession and not directly affecting boarding reduction. This position paper proposes that the ED crowding problem can be optimally addressed by applying a systems approach using predictive modeling to identify patients at risk of being admitted to the hospital and uses that information to initiate the time-consuming bed management process earlier in the care continuum, shortening the time during which patients wait in the ED for an inpatient bed assignment, thus removing the exit block that causes boarding and subsequently reducing crowding.

## Introduction

### The Emergency Department Crowding Problem

The ED crowding problem occurs when the ED demand exceeds the staff’s ability to provide quality care in a reasonable period of time [1,2]. The literature suggests that hospital exit block [3,4] (ie, when patients cannot transition into the hospital from the ED because a hospital bed has not been assigned [3]) and ED boarding [5-8] (ie, when a patient due to be admitted to the hospital remains in the ED, occupying a bed [3]) are the main causes of ED crowding and posits that an impactful solution lies in changes in the bed management strategy, the processes involved in the transition of patients from the ED to the hospital, and when securing a hospital bed [9,10].

This position paper proposes that the complexities of the ED crowding problem can be optimally addressed by applying a systems approach to the hospital bed management strategy. The systems approach views an environment as a whole, which is made up of many parts or subsystems for the purpose of understanding the relationships between the system and its parts and to aid in problem-solving [11]. A systems approach that uses predictive modeling to identify patients who are at risk of being admitted to the hospital and uses that information to initiate the complex and time-consuming bed management process earlier in the care continuum could potentially shorten the time during which patients wait in the ED to be transitioned into an inpatient bed, thus removing the exit block that causes boarding and subsequently reducing crowding [12]. The challenges facing the health care industry are greater than ever before, with increasing complexities of care, regulations, and higher quality care expectations. At the same time, the industry is challenged to address preventable medical errors, poor amenable mortality rates, nursing and physician burnout and shortages, and general inefficiencies. These industry challenges are magnified in the ED where the nature and environment of emergency care cannot tolerate threats to quality care delivery or to patient and provider safety. A systems approach views the ED as a complex microcosm of a larger health ecosystem where optimal functionality requires that it be resilient to the unpredictable demands [13] characteristic of the urgent care environment. To manage new and unpredictable challenges, a systems approach can be used to address known threats. An efficient manner by which to accomplish this is to identify predictable and repeatable processes to which information technology can be applied.

Crowding and its main causes, exit block and boarding, have threatened the quality and safety of ED care for over 20 years despite the efforts of many to resolve it [14-16]. Boarding compromises care quality [3,17], stresses hospital operations [18], and strains resources because boarded patients occupy beds and divert staff resources from new and existing patients [17] and reduce revenue generation through the reduction in available beds for treating new patients [19].

The industry would benefit from improved approaches to resolve the crowding problem. The existing research focused on this problem suggests that predictive modeling holds promise to make a significant contribution toward addressing ED crowding; for example, models have predicted imminent hospital admissions for older ED patients [20], identified patients who are likely to require hospital care in the future [21], predicted ED crowding using calendar and weather variables [22], and forecasted ED flow digitally [23].

### Applying Predictive Modeling to Resolve Crowding and Bed Management

Predictive modeling is a form of data mining technology that functions by analyzing historic and current data, and generating a model to help predict a future outcome [24]. It is an explicit, empirical approach for estimating the probabilities that an event will or will not occur in the future [25]—such as death, contracting a disease, surgical complications, or hospital admission—by using statistical techniques to predict future events. Models use data about patients, diseases, or treatment characteristics to estimate the probability that a condition or disease is present or the probability that an outcome will occur [26,27]. However, models are only just starting to be used to produce actionable information to impact operations and patient care. We posit that predictive models to identify patients who are at risk of being admitted could be applied in the bed manager environment to remove the exit block that causes boarding by initiating the bed management process earlier in the care continuum, thereby shortening the time during which patients wait in the ED for an inpatient bed assignment. This, in turn, reduces exit block, boarding, and subsequently crowding. As no clinical decisions are made on the basis of patients’ risk of admission, this process could be automated to streamline part of the complicated bed management process and take advantage of predictable and repeatable processes using standardized data.

Many interventions to reduce crowding have not been comprehensive or system solutions but rather focus on part of the care procession and do not directly affect boarding reduction [28]. However, existing interventions that addressed crowding as a systemic problem have reduced the time during which a patient is boarded in the ED [9,10,29], which, in turn, reduces the backlog of boarded patients who contribute to ED crowding. Interdepartmental collaboration with hospital management support was a feature in these interventions. Two of these interventions also used real-time ED data on congestion, flow, and patient admissions to prepare for and manage inpatient admissions and bed demand [9,29]. Individual interventions are parts of the system, rather than being considered a collective, and are automated to contribute valuable data to augment bed management.

The use of predictive models in health care have quadrupled over the last 2 decades and their accuracy has increased [12,28]. While these have traditionally been applied to identify risk, the time is right for integrating predictive models with existing technologies such as electronic health records, clinical decision support systems, and clinical data warehouses, to result in action and efficiencies. n 2019, the use of predictive modeling was reported among 60% of health care executives within their organizations, and another 20% of them expressed intent to begin using them the following year [30], again primarily for risk prediction and not necessarily action. This existing technological infrastructure holds promise to reduce prior barriers to integration. Moreover, as clinical care becomes increasingly personalized, aid from predictive analytics may become a best-practice procedure.

### Concerns for Risk Models

Developing quality predictive models is challenging [28,31]. Deciding what variables to measure to answer a particular question can even be problematic. For example, if the goal is to predict “health,” which data are measured as indicators of health? The answer to this question varies. If rigorous study methods are not used throughout study development, data gathering, and analysis, there are numerous avenues for the model to make errors and lead to unintentional bias. Recent studies, including a systematic review of admission prediction models [28], have questioned the quality and rigor of existing predictive models [12,28,31-33] due to an overall lack of external validation studies [31], multiple prediction models for the same outcome or target population [26], and risks of bias [34-36]. A bias unique to predictive models, algorithmic bias, occurs when technology reflects the attitudes and values of the humans who coded, collected, selected, or used the data to train the algorithm [37]. Thus, machine-generated algorithms are human products executed by a machine. Algorithms should not be blindly trusted or considered neutral and unbiased [37]. Reliance on an algorithm to predict health-related outcomes or to make decisions about care would increase the pace of decision-making, but the point at which the decision should be transferred from machine to human is necessary, unclear, and currently unregulated [37].

## Conclusions

Large amounts of data are available for analysis, and the demands on the health care industry are increasing, making the use of predictive modeling to aid hospital operations sensible and increasingly necessary. The old adage “garbage in garbage out” remains true when applied to predictive models—models developed without quality methodologies risk producing predictions deficient of quality. A model that produces biased predictions may not resolve the problem at hand. Evidence that a model is effective and safe is necessary before its use in a clinical setting. Best practices promoting standards for development and operation will have a role to play in model improvement and their use in clinical settings.

Application of models that predict hospital admission could aid hospital bed managers to secure an appropriate bed for a patient in a timely manner while boosting hospital efficiency and with no harm to patients. The result of this timely and streamlined systems process is better patient care delivered sooner.

We posit that applying a systems approach using prediction models to the hospital bed management strategy for ED patients would reveal the many parts and subsystems involved before and after bed assignment and would ensure that they are part of the solution. This unique application of a prediction model provides bed managers information to support initiation of bed management processes earlier in the care continuum. This strategic use of information has significant potential to reduce hospital exit block and ED boarding, and subsequently ED crowding [12].

## Abbreviations

ED: emergency department
